# Circulating Long Non-Coding RNAs as a Promising Non-Invasive Tool to Trace Adiposity Capacity Following Obesity Surgery

**DOI:** 10.3390/life16050727

**Published:** 2026-04-25

**Authors:** Kazim Senol, Secil Ak Aksoy, Gulcin Tezcan, Cagla Tekin, Melis Ercelik, Murat Ferhat Ferhatoglu, Ebru Vatansever, Aysen Akkurt Kocaeli, Berrin Tunca

**Affiliations:** 1 Department of General Surgery, Faculty of Medicine, Bursa Uludag University, 16059 Bursa, Turkey; 2 Department of Medical Microbiology, Faculty of Medicine, Bursa Uludag University, 16059 Bursa, Turkey; 3 Department of Fundamental Sciences, Faculty of Dentistry, Bursa Uludag University, 16059 Bursa, Turkey; 4 Department of Medical Biology, Faculty of Medicine, Bursa Uludag University, 16059 Bursa, Turkey; 5 Department of General Surgery, Faculty of Medicine, Okan University, 34959 Istanbul, Turkey; 6 Department of Biochemistry, Yuksek Ihtisas Hospital, 16310 Bursa, Turkey; 7 Department of Endocrinology, Bursa State Hospital, 16300 Bursa, Turkey

**Keywords:** obesity surgery, sleeve gastrectomy, long non-coding RNA, adiposity

## Abstract

**Highlights:**

**What are the main findings?**

- Plasma expression levels of NEAT1 and HULC are elevated in obese patients but significantly decrease following sleeve gastrectomy.
- Postoperative decreases in NEAT1 and HULC expressions positively correlate with reductions in HbA1C and BMI, respectively.

**What is the implication of the main finding?**

- Time-dependent analysis of serum NEAT1 and HULC can provide valuable insights into weight loss and the resolution of obesity-related comorbidities post-surgery.
- Circulating long non-coding RNAs possess strong potential as a non-invasive tool for tracking adiposity capacity in patients undergoing bariatric surgery.

**Abstract:**

Background/Aim: Long non-coding RNAs (lncRNAs) such as NEAT1, HULC, and MALAT1, which are expressed in adipose tissue, are known to play a role in regulating adiposity. However, how the plasma expression of these lncRNAs changes in obese patients following rapid adipose tissue loss after sleeve gastrectomy remains unclear. This study aimed to investigate the relationship between plasma NEAT1, HULC, and MALAT1 expression levels and short-term weight loss after sleeve gastrectomy. Materials and Methods: Plasma samples prospectively collected from patient groups were used for total RNA extraction to measure the expression levels of NEAT1, HULC, and MALAT1 both before sleeve gastrectomy and 30 days after the procedure. Additionally, patients were followed for changes in body mass index (BMI) and HbA1C levels over a 12-month period. Associations between lncRNA expression levels and clinical parameters were evaluated. Results: Before sleeve gastrectomy, the expression levels of NEAT1 and HULC were significantly higher in obese patients compared to non-obese individuals (*p* < 0.0001). Sleeve gastrectomy was associated with decreased expression levels of NEAT1 (*p* = 0.004) and HULC (*p* = 0.0027). NEAT1 and HULC expression levels showed significant associations with changes in HbA1C and BMI, respectively (*p* < 0.05). Conclusions: NEAT1 and HULC expression levels were associated with short-term metabolic and anthropometric changes following sleeve gastrectomy. These findings are exploratory and hypothesis-generating, and further studies with larger cohorts and longer follow-up are needed to determine their potential clinical relevance.

## 1. Introduction

Almost 13% of human adults suffer from obesity, and 39% of those are described as overweight worldwide [1]. The increased propensity towards metabolic disorders, such as type 2 diabetes, non-alcoholic fatty liver disease (NAFLD), and cardiovascular disease that accompanies obesity, has rendered it a pervasive global health concern [2,3]. According to the World Health Organization (WHO), a healthy individual has a body mass index (BMI) between 18.5 and 24.9, while they are described as overweight with a BMI higher than 25 and considered morbidly obese with a BMI higher than 35 [4]. Contemporary therapeutic approaches for obesity involve lifestyle modifications, such as dietary adjustments, enhanced physical activity, and bariatric surgery [5,6]. Despite the above interventions, a proportion of individuals encounter the recurrence of weight-related comorbidities after treatment [7].

Obesity is a multifactorial disorder influenced by genetic and environmental factors that may contribute to its susceptibility and progression [8,9]. Basic social and environmental factors, such as diet, lifestyle, and stress, can impact biological processes, potentially augmenting fat accumulation, lipogenesis, and adipogenesis via the regulation of long non-coding RNA (lncRNA) expression [10,11]. LncRNAs, transcripts that are longer than 200 nucleotides, involve regulating the transcription and maturation of mRNA in the nucleus and modulating the stability and functionality of mRNA and miRNA in the cytoplasm, which results in changes in the expression and function of proteins involved in specific signaling pathways [12,13]. Recent studies have elucidated the pivotal regulatory role of lncRNAs in the signaling pathways of inflammation, adipogenesis, and lipid accumulation [14,15,16]. Overexpression of lncRNA, Nuclear enriched abundant transcript 1 (NEAT1), has been associated with accelerated obesity and NAFLD via increased lipid accumulation, adipogenesis, and the promotion of fibrosis formation [15,17,18,19,20]. The overexpression of hepatocellular carcinoma upregulated long non-coding RNA (HULC) has been found to impede lipid metabolism and elevate intracellular levels of triglycerides and cholesterol in liver-related pathologies, including NAFLD [21,22,23,24]. Furthermore, high expression levels of Metastasis-associated lung adenocarcinoma transcript 1 (MALAT1) have been demonstrated to promote the production of inflammatory cytokines, insulin resistance, lipogenesis, and adipogenesis in microvascular diseases associated with diabetes [25,26]. As NEAT1, HULC, and MALAT1 are known to target adipogenesis, their dysregulation is believed to play a role in the predisposition to obesity [15,23,26]. Considering the genetic and lifestyle differences amongst individuals with obesity, it is arguable that the expression pattern of NEAT1, HULC, and MALAT1 may serve as a predictive marker for obesity resolution following bariatric surgery and disease recurrence in the future. In this context, before an invasive surgical procedure, it would be valuable to anticipate the benefit to be gained from treatment with a non-invasive method. Previous studies determined the expression pattern of NEAT1, HULC, and MALAT1 based on their cellular expressions [15,23,26]. However, the diagnostic value of the plasma lncRNA signature for obesity predisposition remains unknown. In this study, we focused on NEAT1, HULC, and MALAT1 based on their well-established mechanistic roles in adipogenesis, lipid metabolism, and inflammation, as these lncRNAs have been consistently implicated in obesity-related pathways across multiple studies. We designed a hypothesis-driven investigation to assess whether the plasma expression of these specific lncRNAs could serve as non-invasive markers for obesity-related metabolic changes and to evaluate their potential predictive value for weight regain following bariatric surgery.

## 2. Materials and Methods

### 2.1. Patient Cohort

A total of 20 obese patients who underwent laparoscopic sleeve gastrectomy and eight age- and sex-matched healthy volunteers were enrolled in this observational cohort study. The obese patients were assessed before and after surgery by a multidisciplinary team, including an anesthesiologist, endocrinologist, pulmonologist, and psychologist, in accordance with international metabolic and bariatric surgery guidelines. The patient’s body mass index (BMI), fasting glucose, and hemoglobin (HbA1C) levels, as well as liver function profile tests (Alanine aminotransferase (ALT), Aspartate aminotransferase (AST)), measured one day before the surgery, were considered as preoperative baseline data (Table 1). Additionally, RNA samples obtained from the plasma of the patients one day before the sleeve gastrectomy and 30 days after surgery were included in the study. The patients’ BMI, fasting glucose, and HbA1C levels were measured one day before the surgery, and the postoperative 1st, 6th, and 12th months were also included in the analysis (Table 2). ΔBMI was defined as the difference between postoperative and preoperative BMI (ΔBMI = BMI_post − BMI_pre).

The control group consisted of eight healthy volunteers who were carefully selected according to stringent inclusion and exclusion criteria to establish a reliable reference baseline for comparative analyses and to enable a preliminary evaluation of differences in circulating biomarkers. Inclusion criteria required a body mass index (BMI) within the normal range (18.5–24.9 kg/m^2^) and the absence of any history of obesity, metabolic syndrome, diabetes mellitus, cardiovascular disease, hepatic or renal dysfunction, malignancy, or recent infections. Individuals receiving regular medications or presenting with any clinical or laboratory evidence of systemic illness were excluded. In fact, comorbidity status was rigorously assessed for all individuals in the control group prior to inclusion. A detailed medical history was obtained from each volunteer, and comprehensive clinical and biochemical evaluations were performed to rule out any chronic or systemic conditions. Participants with known or suspected comorbidities—including but not limited to obesity, diabetes mellitus, hypertension, cardiovascular disease, chronic liver or kidney disease, malignancy, or recent infections—were excluded from the study. All control participants underwent a standard clinical examination and routine biochemical screening to confirm their healthy status before enrollment. The mean age of the control group was 36 years (range: 28–43), compared to 33 years (range: 21–51) in the patient group.

This study was approved by the local Ethics Committee of Okan University (approval number: 08.01.2020/24). All methods were carried out in accordance with relevant guidelines and regulations. Signed informed consent was obtained from all participants.

### 2.2. Real Time-qPCR

Total RNA isolation was performed using the Ambion RNA isolation kit (Thermo Fisher Scientific, Waltham, MA, USA). RNA quantity and quality were assessed using a UV/Vis spectrophotometer (Beckman Coulter, Brea, CA, USA). A total of 500 ng RNA was reverse-transcribed into cDNA using the Biolabs cDNA Synthesis kit (New England Biolabs, Frankfurt am Main, Germany). The cDNA samples were analyzed for the presence and differential expression of adipogenesis and NAFLD-related lncRNA, including NEAT1, MALAT1, and HULC (Hs03453534_s1; Hs03453854_g1 and Hs01909631_s1; Thermo Fisher, CA, USA). The RNA input of LncRNA expressions was normalized to a housekeeping gene, GAPDH (Hs02786624_g1; Thermo Fisher, CA, USA). The reaction was performed as follows: an initial Uracil-N-Glycosylase (UNG) incubation at 50 °C for 2 min, followed by polymerase activation at 95 °C for 10 min, and then 40 cycles of denaturation at 95 °C for 15 s and annealing/extension at 60 °C for 60 s. The threshold cycle (Ct) for RNA expressions was determined using the Applied Biosystem StepOne^TM^ RT-PCR Software v2.3 (Thermo Fisher, CA, USA). The fold changes between expression levels were calculated using the 2^−ΔΔC(T)^ method [27]. RNA input amounts were standardized and reported in mass units (ng). Quality control criteria were applied prior to analysis. Ct values were evaluated according to predefined thresholds, and samples with inconsistent amplification were excluded. All measurements were performed in technical replicates, and average Ct values were used for downstream analysis.

### 2.3. Statistical Analysis

Normality of the data was assessed using the Shapiro–Wilk test. As the data were not normally distributed, non-parametric tests were used for all analyses.

Comparisons between independent groups (patients with obesity vs. healthy controls) were performed using the Mann–Whitney U test, while paired comparisons (preoperative vs. postoperative samples) were analyzed using the Wilcoxon signed-rank test. The Mann–Whitney U test was also used to examine the impact of comorbidities on lncRNA expression levels.

Correlation analyses were primarily performed using Pearson correlation to assess linear relationships. In addition, Spearman rank correlation was conducted as a sensitivity analysis to confirm the robustness of the results.

All statistical analyses and graph plotting were performed using GraphPad Prism version 8.0 (GraphPad Software Inc., San Diego, CA, USA). A *p*-value of <0.05 was considered statistically significant.

## 3. Results

### 3.1. Clinicopathological Features of Patients

**Figure 1:**
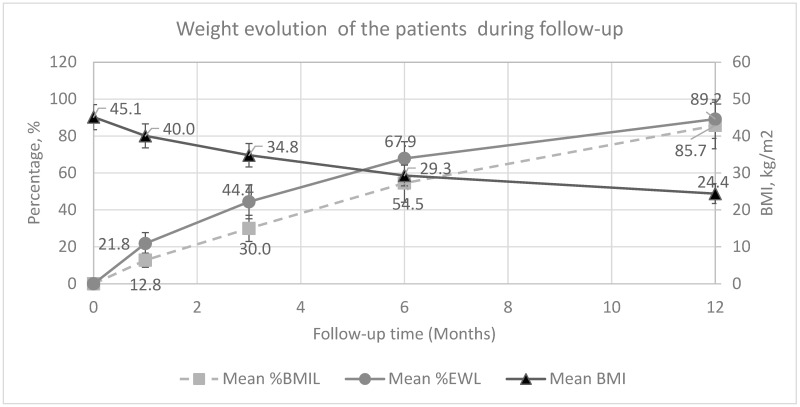
Weight loss evolution of the patients during 1-year follow-up. (Excess weight loss percentage-EWL% and BMI loss (secondary *y*-axis), BMI loss percentage-BMIL%).

The median age of the patients was 33 years (Range: 21–51), whereas it was 36 years (Range: 28–43) for healthy volunteers. The preoperative median BMI of obese patients was 44.2 ± 0.7 kg/m^2^ (Range: 39–54). Three patients (15%) had type-2 diabetes, two patients (10%) had hypertension, and four patients (%20) had other co-morbid diseases, including obstructive sleep apnea, asthma, hypothyroidism, hyperlipidemia, and polycystic ovary syndrome. Laparoscopic sleeve gastrectomy was performed in all of the patients. There were no per- and post-operative complications. The median hospital stay was 4 days (Range: 3–9), and all of the patients were discharged from the hospital uneventfully. Weight loss evolution (EWL% and BMIL) during 12 months of follow-up is mentioned in Figure 1.

### 3.2. Sleeve Gastrectomy Led to a Change in the Expression Levels of LncRNA in Obese Patients

**Figure 2:**
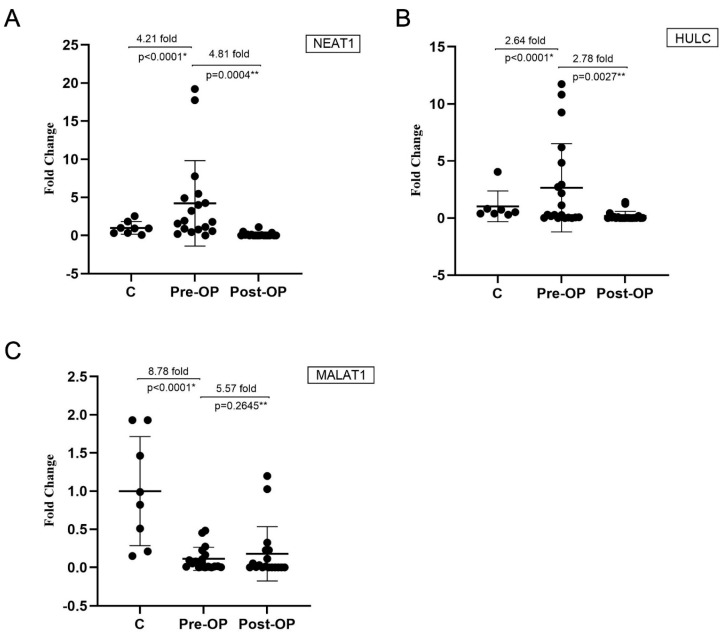
Changes in lncRNA expression levels before and after sleeve gastrectomy in obese patients compared with controls. (**A**) NEAT1, (**B**) HULC, (**C**) MALAT1. C: control; Pre-OP: before sleeve gastrectomy; Post-OP: 1 month after sleeve gastrectomy. Relative expression levels were calculated using the 2^−ΔΔCt^ method. Data are presented as median (min–max). Comparisons between control and preoperative groups were performed using the Mann–Whitney U test, while paired comparisons between preoperative and postoperative samples were analyzed using the Wilcoxon signed-rank test. *p*-values are unadjusted. * *p* < 0.05, ** *p* < 0.01. Statistical comparisons were performed as follows: control vs. preoperative (independent), preoperative vs. postoperative (paired).

This analysis included 20 obese patients and 8 healthy controls. One day prior to sleeve gastrectomy, plasma NEAT1 expression levels in obese patients were significantly elevated, with a 4.21 ± 1.32-fold increase compared to non-obese individuals (*p* < 0.0001, Cohen’s d = 0.43, power = 47%; Figure 2A). HULC expression was 2.64 ± 0.86-fold higher in obese patients compared to healthy controls (*p* < 0.0001, Cohen’s d = 0.47, power = 48%; Figure 2B). In contrast, MALAT1 expression was markedly lower in obese patients, showing an 8.78 ± 0.03-fold reduction relative to healthy controls (Cohen’s d = 2.18, power > 99%; Figure 2C).

Thirty days after sleeve gastrectomy, NEAT1 expression levels decreased significantly by 4.81 ± 0.06-fold (*p* = 0.004), and HULC expression dropped by 2.78 ± 0.09-fold (*p* = 0.0027), indicating a robust early postoperative downregulation (Figure 2A,B). Conversely, MALAT1 expression showed no significant change postoperatively (*p* = 0.2645; Figure 2C). These findings suggest that sleeve gastrectomy selectively modulates specific lncRNAs in the early postoperative period. The significant reduction in NEAT1 and HULC expression—both of which were elevated preoperatively—suggests a potential role for these lncRNAs in the metabolic adaptations following bariatric surgery.

### 3.3. Post-Operative Decrease in Body Mass Index Was in Correlation with NEAT and HULC Expressions

**Figure 3:**
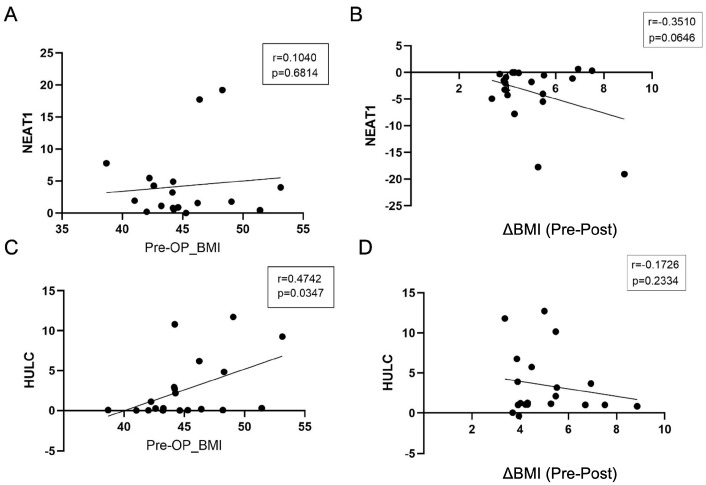
Correlation analyses between BMI parameters and lncRNA expression levels. (**A**) Correlation between preoperative BMI and NEAT1 expression. (**B**) Correlation between ΔBMI and NEAT1 expression. (**C**) Correlation between preoperative BMI and HULC expression. (**D**) Correlation between ΔBMI and HULC expression. ΔBMI was calculated as postoperative BMI minus preoperative BMI (ΔBMI = BMI_post − BMI_pre). The y-axes represent relative expression levels of NEAT1 and HULC. Correlations were assessed using Pearson correlation analysis; similar results were obtained using Spearman correlation *p* < 0.05.

Correlation analyses were conducted on data from 20 obese patients who underwent sleeve gastrectomy. Before surgery, Pearson correlation analysis revealed a weak and non-significant positive correlation between NEAT1 expression levels and BMI (r = 0.1040; *p* = 0.6814; Figure 3A). In contrast, a moderate positive correlation with statistical significance was observed between HULC expression levels and BMI (r = 0.4742; *p* = 0.0347; Figure 3C), indicating that higher baseline HULC expression was associated with higher BMI values. Similar results were obtained using Spearman correlation analysis.

At 30 days post-surgery, we assessed the relationship between ΔBMI and lncRNA expression levels. Both NEAT1 and HULC showed negative correlations with ΔBMI, indicating that greater weight loss was associated with lower expression levels (NEAT1: r = −0.3510; *p* = 0.0646; HULC: r = −0.1726; *p* = 0.2334; Figure 3B,D). The correlation for NEAT1 approached statistical significance.

Furthermore, we explored whether lncRNA expression levels at 30 days post-surgery were associated with mid- and long-term weight loss outcomes. A weak positive correlation was observed for weight loss between 1 and 6 months after surgery, although this did not reach statistical significance (r = 0.2074; *p* = 0.1901). Similarly, a weak positive correlation persisted between 6 and 12 months (r = 0.2109; *p* = 0.1861). For HULC, expression levels at 30 days post-surgery showed weak positive correlations with weight loss at both 6 months (r = 0.05053; *p* = 0.4162) and 6–12 months (r = 0.3203; *p* = 0.0843).

These findings suggest that NEAT1 and HULC expression levels may be associated with short-term weight loss patterns; however, their potential role in predicting long-term outcomes requires further investigation.

### 3.4. Comorbidities Did Not Affect the Regulation LncRNA Expressions

**Figure 4:**
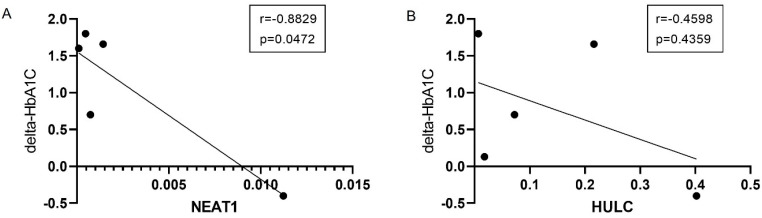
The relationship between change in HbA1C after surgery and change in LncRNA expression (**A**) NEAT1, (**B**) HULC. delta_HbA1C: The rate of change observed in HbA1C before and after surgery. *p* values were calculated using a Mann–Whitney Test *p* < 0.05.

We further assessed whether the presence of comorbidities had any impact on lncRNA expression levels. Specifically, we compared NEAT1 and HULC expression levels between patients with at least one comorbidity and those without comorbidities, using samples collected one day before sleeve gastrectomy. No significant differences were found between the two groups for either NEAT1 (*p* = 0.3749) or HULC (*p* = 0.8168) expression (Figure 4A,B), suggesting that baseline expression levels of these lncRNAs are not markedly influenced by the presence of comorbid conditions.

**Figure 5:**
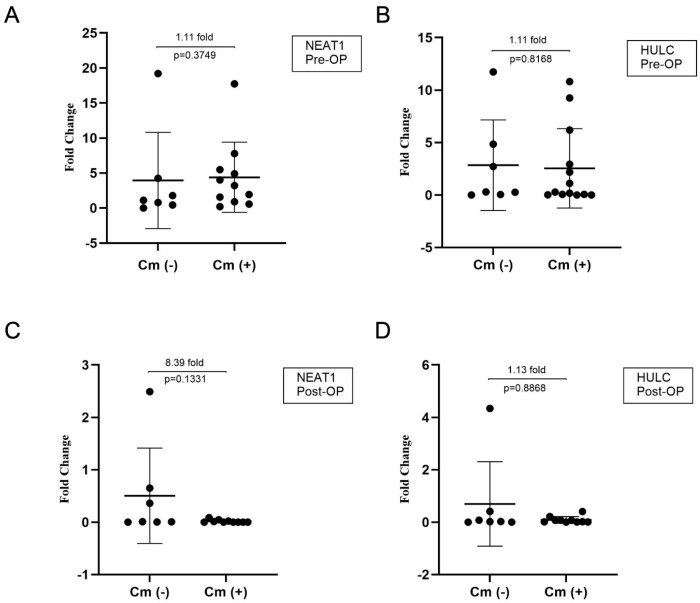
Comparison of NEAT1 and HULC expression levels according to comorbidity status before and after sleeve gastrectomy. (**A**) NEAT1 expression levels in the preoperative period. (**B**) HULC expression levels in the preoperative period. (**C**) NEAT1 expression levels in the postoperative period (30 days). (**D**) HULC expression levels in the postoperative period (30 days). Cm: comorbidity; Cm (−): no comorbidity; Cm (+): presence of one or more comorbidities. Pre-OP: before sleeve gastrectomy; Post-OP: 30 days after sleeve gastrectomy. The y-axes represent relative expression levels. Data are presented as mean ± SD. Statistical comparisons between groups were performed using the Mann–Whitney U test. *p* < 0.05.

In addition, we examined whether comorbidity status was associated with lncRNA expression levels following surgery. No significant differences were observed between patients with and without comorbidities for NEAT1 (*p* = 0.1331) or HULC (*p* = 0.8868) in the postoperative period (Figure 5C,D). Similarly, no significant differences were detected between groups in the preoperative expression levels (Figure 5A,B). These findings suggest that comorbidity status was not significantly associated with NEAT1 and HULC expression levels in this cohort.

## 4. Discussion

Sleeve gastrectomy has been described as a standalone, single-stage procedure in the surgical management of obesity with favorable outcomes regarding enhanced quality of life and resolution of comorbidities [28]. Studies have shown that patients typically lose an average of 60–70% of their excess weight within the first year following the surgery [29]. The weight loss after a sleeve gastrectomy can vary depending on several factors, including the individual’s physiology molecular and genetic alterations in glucose and lipid metabolism. The functions of lncRNAs include epigenetic, transcriptional, and post-transcriptional regulation, as well as induction of gene expression through their ability to act as either cis- or trans-regulatory elements [30]. Furthermore, clinical and *in vitro* analyses have demonstrated that sleeve gastrectomy upregulates lncRNA TUG1, which subsequently protects intestinal epithelial cells from high glucose and high fat-induced damage by modulating the AMPK/SIRT1/UCP2 signaling axis; this mechanism is proposed to play a crucial role in the therapeutic efficacy of bariatric surgery for type II diabetes [31]. A recent animal study demonstrated that hepatic lncRNA Gm19619, which is enriched along genomic regions encoding leptin receptors, contributes to the metabolic effects of sleeve gastrectomy in mice by promoting hepatic gluconeogenesis and lipid accumulation [32]. These findings suggest that lncRNAs may enhance gluconeogenesis and lipid accumulation in both liver and intestinal cells. Owing to their varied and complex roles in cellular processes, the investigation of lncRNAs in adipogenesis and glucose metabolism remains an active field of research.

Previous studies reported the involvement of NEAT1, HULC, and MALAT1 in the regulation of adipogenesis [33,34], which is the critical process to increase the number of adipocytes and lipid-storing capacity of adipose tissue and fat mass [35]. Therefore, the rationale for selecting the lncRNAs NEAT1, HULC, and MALAT1 in this study lies in their known roles in adipose and hepatic metabolism, as well as their potential to acutely respond to postoperative metabolic changes [36,37,38]. In support of this, our findings showed enhanced expression levels of NEAT1 and HULC in the plasma of obese patients. In addition, the expression levels of these LncRNAs were sharply reduced after sleeve gastrectomy. These findings suggest that LncRNA NEAT1 and HULC may be released to peripheral blood from adipose tissues.

Conversely, although the role of MALAT1 in adipogenesis through the regulation of PPAR-γ transcription has been previously described [39], our findings revealed low levels of MALAT1 in the plasma of obese individuals. In addition, sleeve gastrectomy did not cause a measurable change in MALAT1 expression within 30 days post-surgery, suggesting that adipose tissue-derived MALAT1 may not be detectably released into the peripheral plasma during this period. However, the long-term effects of sleeve gastrectomy on serum MALAT1 levels and its potential predictive value remain open to investigation.

A positive correlation between NEAT1 overexpression and BMI was previously demonstrated in osteoarthritis [40], while a similar association could not be defined in acute ischemic stroke [41]. Therefore, the current knowledge on the association between NEAT1 and BMI is controversial and may depend on the underlying disease. In addition, our understanding of the relationship between HULC and BMI is mainly unknown. Based on our pilot findings, the level of fatty cell-secreted NEAT1 and HULC correlates with BMI. While the weight loss results in loose fatty cells, it also causes a decrease in NEAT1 and HULC in peripheral serum due to increased adipocyte destruction.

In addition to adipogenic capacity, NEAT1 has been shown to promote inflammasome formation in macrophages, leading to increased IL-1β secretion, suggesting its capacity to rapidly trigger inflammatory responses in immune cells [42]. In mouse models, NEAT1 deficiency has been associated with attenuated inflammatory responses and reduced hepatic lipid accumulation upon silencing [42,43]. In our study, findings obtained within the first 30 days after bariatric surgery suggest that NEAT1 may act as an early modulated mediator in the adipocyte cycle. These mechanistic insights indicate that early NEAT1 expression levels could play a critical role in adipose tissue remodeling and the reduction in inflammation in the early postoperative period.

In the study by Cui et al. (2015), it was demonstrated that HULC enhances the expression of PPARα and its target gene, ACSL1 by suppressing miR-9, thereby accelerating triglyceride and cholesterol synthesis [23]. Moreover, studies in animal models have shown that HULC plays significant roles in the pathogenesis of non-alcoholic fatty liver disease (NAFLD) and liver fibrosis [21,44,45]. Additionally, it has been reported that HULC is required for a full inflammatory response during endotoxemia, supporting its involvement in inflammation-related mechanisms [46]. All these findings in the literature suggest that overexpression of HULC increases intracellular lipid accumulation. Furthermore, inhibition of HULC has been shown to reverse this effect [23]. In line with these reports, our study also demonstrated a significant decrease in HULC levels on postoperative day 30 following bariatric surgery. When evaluated in the context of the existing literature, our findings suggest that hepatic lipid mobilization may begin early after surgery and that HULC could serve as one of the early molecular markers of this process.

Although there is scarce data regarding the current study’s design in the literature, recent transcriptomic studies reveal that sleeve gastrectomy leads to significant molecular changes linked to metabolic improvement. Across three independent studies, the effects of sleeve gastrectomy on inflammatory signaling, immune responses, and lncRNA regulation were consistently highlighted. In the study by Liu et al. [36], bulk RNA-seq analysis of peripheral blood mononuclear cells (PBMCs) revealed notable changes in gene expression one month after sleeve gastrectomy in obese Chinese patients. The study identified three key genes (IRF1, NFKBIA, YRDC) whose expression changes surgery correlated with immune and lipid metabolism pathways, along with reduced leptin levels and lncRNA-mediated regulation. Complementing this, Poitou et al. [37] conducted RNA-seq analysis on subcutaneous adipose tissue from 22 obese women before and three months after surgery. They identified 1214 differentially expressed genes with significant transcriptional downregulation in inflammatory and immune-related pathways. The authors demonstrated that sleeve gastrectomy disrupts immune signaling networks in adipose tissue, particularly T-cell and neutrophil-mediated inflammation, suggesting that early metabolic benefits may result from attenuated immune activity. Further supporting these observations, Tokgun et al. [38] analyzed serum lncRNA expression in 15 obese patients before and six months after sleeve gastrectomy. The expression of eight lncRNAs (H19, Neat1, HOTAIR, ANRIL, MALAT1, ATB, SNGH5, UCA1) significantly increased following surgery, and their levels correlated with reductions in BMI and metabolic parameters. These lncRNAs are implicated in pathways regulating adipogenesis, lipid metabolism, and insulin resistance. Particularly, lncRNAs like H19 and MALAT1, previously linked to adipocyte differentiation and obesity-related diseases, may be promising biomarkers for monitoring metabolic response post-surgery. These studies demonstrate that sleeve gastrectomy induces widespread transcriptomic remodeling in systemic immune cells and local adipose tissue, accompanied by lncRNA expression changes that reflect metabolic improvement.

This study has several limitations. First, the observational design and relatively small sample size may limit statistical power and the generalizability of the findings. Second, multiple correlation analyses were performed without adjustment for multiple testing; therefore, the results should be considered exploratory. Third, the limited number and heterogeneity of comorbid conditions—predominantly diabetes—restricted subgroup analyses. Finally, potential confounding factors such as postoperative medication use, dietary habits, and physical activity were not systematically evaluated. Larger, well-designed prospective studies with more homogeneous cohorts are required to validate these findings and further clarify the role of lncRNAs in metabolic outcomes following sleeve gastrectomy.

## 5. Conclusions

This study demonstrates that plasma levels of the lncRNAs NEAT1 and HULC are reduced 30 days after sleeve gastrectomy and are associated with short-term metabolic and anthropometric changes. These findings provide preliminary evidence that lncRNA expression patterns may reflect early postoperative responses; however, they should be interpreted as exploratory and hypothesis-generating rather than predictive.

Further studies in larger, well-characterized cohorts with longer follow-up are required to determine the potential clinical relevance of these findings. In addition, mechanistic studies using in vitro and in vivo models are needed to better understand the role of NEAT1 and HULC in adipogenesis and metabolic regulation. The application of high-throughput approaches, such as RNA sequencing, and the integration of plasma and tissue-level data may further clarify the biological significance of these lncRNAs.

## Figures and Tables

**Table 1:** Preoperative demographic characteristics of obese patients and healthy controls involved in the study.

Variable	Patients, n = 20	Control n = 8
Age, years	33 (21–51)	36 (28–43)
Gender, Female, n (%)	11 (55%)	4 (50%)
Body mass index (kg/m^2^)	44.23 (39.0–58.0)	21.32 (19.78–23.56)
Comorbidities, count (%)		-
One	9 (45%)	-
Two or more	11 (55%)	-
Fasting plasma glucose, (mg/dL)	121 (92–168)	80 (76–88)
HbA1C (%)	7.1 (5.3–15.0)	-
Total cholesterol level, (mg/dL)	225 (98–335)	
ALT (U/L)	35 (7–61)	-
AST (U/L)	21 (12–56)	-

**Table 2:** Postoperative Clinical Characteristics of Obese Patients.

Variable	Patients, n = 20
Hemoglobin A1C level, (%)	
Postoperative 1st month	6.2 (min: 4.8; max: 7.5)
Postoperative 6th month	5.4 (min: 4.32; max: 8.3)
Postoperative 12th month	5.3 (min: 4.67; max: 6.40)
Body mass index (kg/m^2^)	
Postoperative 1st month	39.84 (min: 24; max: 48)
Postoperative 6th month	29.32 (min: 24; max: 34)
Postoperative 12th month	24.41 (min: 20; max: 31)
Excess weight loss %	
Postoperative 1st month	19.89 (min: 14; max: 34)
Postoperative 6th month	68.02 (min: 53; max: 81)
Postoperative 12th month	90.46 (min: 65; max: 104)

## Data Availability

The research data supporting the results of this manuscript are available upon request. Data generated and analyzed during this study are not publicly available due to ethical restrictions or privacy concerns but can be made available from the corresponding author upon reasonable request.
